# Better living through conifer removal: A demographic analysis of sage-grouse vital rates

**DOI:** 10.1371/journal.pone.0174347

**Published:** 2017-03-23

**Authors:** John P. Severson, Christian A. Hagen, Jason D. Tack, Jeremy D. Maestas, David E. Naugle, James T. Forbes, Kerry P. Reese

**Affiliations:** 1 Department of Fish and Wildlife Sciences, University of Idaho, Moscow, Idaho, United States of America; 2 Department of Fisheries and Wildlife, Oregon State University, Bend, Oregon, United States of America; 3 Wildlife Biology Program, University of Montana, Missoula, Montana, United States of America; 4 United States Department of Agriculture, Natural Resources Conservation Service, Redmond, Oregon, United States of America; 5 Lakeview District, Bureau of Land Management, Lakeview, Oregon, United States of America; Centre for Cellular and Molecular Biology, INDIA

## Abstract

Sagebrush (*Artemisia* spp.) obligate wildlife species such as the imperiled greater sage-grouse (*Centrocercus urophasianus*) face numerous threats including altered ecosystem processes that have led to conifer expansion into shrub-steppe. Conifer removal is accelerating despite a lack of empirical evidence on grouse population response. Using a before-after-control-impact design at the landscape scale, we evaluated effects of conifer removal on two important demographic parameters, annual survival of females and nest survival, by monitoring 219 female sage-grouse and 225 nests in the northern Great Basin from 2010 to 2014. Estimates from the best treatment models showed positive trends in the treatment area relative to the control area resulting in an increase of 6.6% annual female survival and 18.8% nest survival relative to the control area by 2014. Using stochastic simulations of our estimates and published demographics, we estimated a 25% increase in the population growth rate in the treatment area relative to the control area. This is the first study to link sage-grouse demographics with conifer removal and supports recommendations to actively manage conifer expansion for sage-grouse conservation. Sage-grouse have become a primary catalyst for conservation funding to address conifer expansion in the West, and these findings have important implications for other ecosystem services being generated on the wings of species conservation.

## Introduction

The sagebrush (*Artemisia* spp.) biome in western North America has experienced numerous threats since the late 1800s including conversion to agriculture, energy development, altered fire regimes, overgrazing, and invasive species [1–3] resulting in significant reduction and degradation of sagebrush habitat [4]. These losses reduce native wildlife populations, particularly sagebrush obligates [1,5].

Conifer expansion is a primary threat throughout the Great Basin and other sagebrush ecosystems [6,7]. Native, species such as piñon pine (*Pinus* spp.) and juniper (*Juniperus* spp.) were historically restricted to low fuel areas such as rocky outcrops that did not burn frequently. More recently, these woody species have increased 3–10 times in distribution and ~10 times in abundance in portions of the Great Basin [8,9] and currently occupy 18 million ha in the Intermountain West [10]. Factors thought to have contributed to conifer expansion include fire suppression, fire fuel reduction via over-grazing, changing climate patterns, and increases in atmospheric CO_2_ [8,11,12]. Increased conifer abundance fragments and displaces sagebrush habitat, provides avian predator perches, and degrades range condition [7,8,13–15].

Greater sage-grouse (*Centrocercus urophasianus*; hereafter, sage-grouse) were once widespread throughout western North America but, as an obligate sagebrush species, has declined along with its requisite habitat [3]. Sage-grouse distribution has contracted 44% since the late 1800s [16], and some populations continue to decline within remaining habitat [17]. Conifer encroachment is thought to be an important threat to sage-grouse, but experimental field studies are limited [2,18]. Reduced sage-grouse lek (i.e. communal breeding ground) occupancy and survival with increasing conifer abundance have been documented and used to infer benefits of management [19,20]. In general, conifer removal can prevent displacement and fragmentation of sagebrush systems, reduce available avian predator perches and nesting habitat, and improve range conditions [8,21–23], but it is not confirmed that sage-grouse benefit from these treatments and determine the time frame of the response.

A key step in ecological restoration is monitoring management outcomes [24,25], and experimentally evaluating restorative actions is an important step in understanding their efficacy [26]. Even more rare is monitoring demographics of the focal species after habitat restoration [27]. Conifer removal is recommended to further sage-grouse conservation [20,28], but little research has investigated sage-grouse response to conifer management. Two of the most important vital rates affecting sage-grouse population growth are annual female survival and nest survival, and managing these parameters should be a focus of sage-grouse conservation [29].

Using a before-after-control-impact (BACI) framework [30,31], we compared female sage-grouse survival and nest survival before and after conifer removal within treatment and control areas from 2010 to 2014. Because conifer removal may increase high quality habitat availability and limit avian predator distribution and abundance, we predicted: 1) female survival would increase after conifer removal with the greatest improvement during nesting and early summer/brood rearing seasons when females are near conifers and susceptible to predation [19,32], and 2) increased nest survival after conifer treatments.

## Methods

### Study area

**Fig 1:**
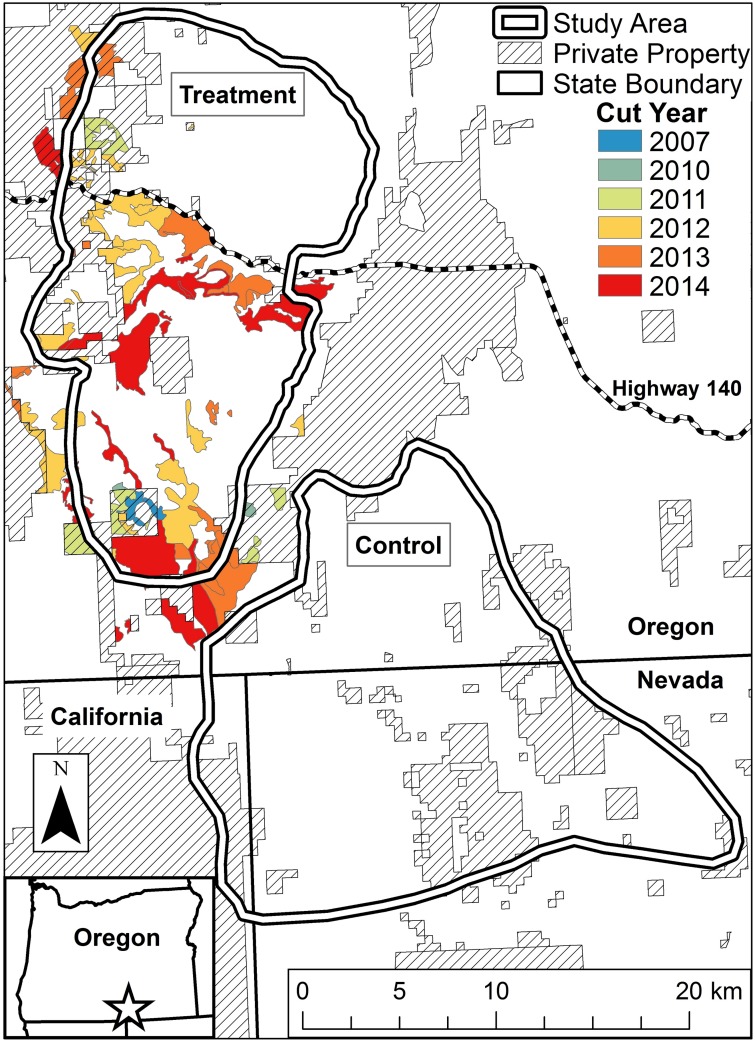
Treatment and control study areas used to assess greater sage-grouse demographic response to conifer management.

Data were collected in a treatment area in southern Lake County, Oregon between the Warner Mountains and the Warner Valley and a control area in southern Lake County south of Warner Valley extending into Modoc County, California north of Cowhead Lake and into Washoe County, Nevada north of Mosquito Lake (Fig 1). We delineated discrete boundaries for treatment and control study areas guided by natural barriers (e.g., canyons, cliffs, and forest) as well as observed sage-grouse movements (Fig 1). The treatment area encompassed 34,000 ha with an average elevation of 1770 m (range: 1490–2100 m) above sea level. The control area encompassed 40,000 ha with an elevation average of 1680 m (range: 1360–2180 m) above sea level. The estimated average annual precipitation from 2010–2014 was 27.7 cm (5.4 SD) in the treatment area and 26.8 cm (5.9 SD) in the control area (Prism Climate Group, prism.oregonstate.edu). The estimated average annual temperature from 2010–2014 was 7.5°C (0.6 SD) in the treatment area and 7.4°C (0.7 SD) in the control area (Prism Climate Group, prism.oregonstate.edu). Both areas were dominated by low sagebrush (*A*. *arbuscula*), but other dominant species included mountain big sagebrush (*A*. *tridentata* ssp. *vaseyana*) at higher elevations (~>1,700 m), Wyoming big sagebrush (*A*. *t*. ssp. *wyomingensis*) at lower elevations (~<1,500 m), and other interspersed shrubs including antelope bitterbrush (*Purshia tridentata*), rabbitbrush (*Chrysothamnus* spp.), saltbrush (*Atriplex* spp.), and mountain mahogany (*Cercocarpus* spp.). Western juniper occurred in patchy distributions from mid to high elevation ($\bar{\text{x}}$: 1,810; 95% distribution: 1,460–2,160 m).

### Conifer management

**Fig 2:**
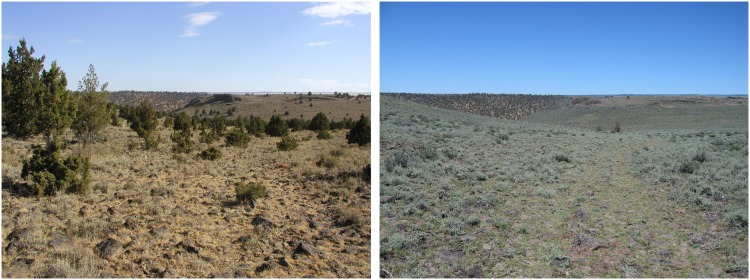
Typical example of conifer-encroached site before tree removal in 2008 (left) and after (right, 2015) in the treatment area, Lake County, OR (photo credit Todd Forbes, Bureau of Land Management).

The Bureau of Land Management (BLM) removed juniper on public land while the Natural Resources Conservation Service in association with the Oregon Department of Fish and Wildlife assisted landowners with juniper removal on private land within and surrounding the treatment area (Fig 1). Treatments generally occurred from late fall to early spring and were designed to maximize shrub retention. Conifer encroachment has been classified into three successional phases: Phase-I is early successional with conifers subdominant in the plant community; Phase-II is mid-successional with conifers codominant in the community; Phase-III is late-successional with conifers dominant in the community [8]. Most of the treated areas were Phase-I to Phase-II encroachment with generally intact understory herbaceous and shrub communities having similar composition and abundance to areas without conifer encroachment (Fig 2). Most treatments (95%) were conducted during late fall to early spring by hand-cutting with brush- and chainsaws to minimize direct negative affects to grouse breeding activities; however, 444 ha were machine cut (e.g., feller-buncher) in fall 2013 to spring 2014. Additional slash treatment of cut conifers was conducted where necessary to reduce woody fuels and vertical structure. Treatments were implemented depending on tree size and density, understory, and landowner preference [on private land] but mostly consisted of cut-leave, cut-lop, cut-burn, and cut-pile-burn. Cut-leave involved cutting trees without additional slash treatment and generally occurred in areas with trees of low size and density. Cut-lop consisted of felling trees and removing tall branches from tree boles to reduce vertical structure and avian predator perches. Cut-burn occurred with larger, denser trees to expose the understory and encourage growth. Generally, cut trees were left to dry for ~1 year and then burned individually. Effort was made to burn only individual trees to reduce shrub mortality and burn scars to minimize negative affects of treatments to beneficial habitat. Cut-pile-burn involved felling trees, cutting into manageable pieces, and stacking in small piles for burning when soils were frozen. This technique was used less often due to cost but was deemed necessary in some areas of high tree density to reduce area impacted by slash-burning. Across all treatments, the objective was complete conifer removal, but an attempt was made to leave pre-settlement trees in locations that historically supported juniper, thus some areas still had standing trees after treatment [33]. BLM biologists identified pre-settlement trees using criteria including size, leader growth, crown form, bark, and habitat [8]. For our analysis we grouped all treatments together.

Although some treatments occurred from 2007–2011 (<10%), most occurred from 2012–2014; slash-burning also began in 2012. We used spring of 2012 as the break between before and after treatment. Within the treatment area, 6488 ha of trees were cut and 2277 ha of trees were slash-burned, while 9443 ha and 3540 ha were cut and slash-burned, respectively, in the greater area with an average treatment size of 87 ha (Table 1; Fig 1).

**Table 1:** Annual areal estimates of cut and slash-burned conifer in the treatment study area.

Year	Treatment Area			Greater Treatment Area			Average Size (ha)
	Cut (ha)	Cumulative Cut (ha)	Slash-Burn (ha)	Cut (ha)	Cumulative Cut (ha)	Slash-Burn (ha)	
2007	143	143	—	143	143	—	72
2010	17	160	—	57	200	—	29
2011	432	592	—	781	981	—	71
2012	2073	2665	95	2709	3690	97	68
2013	1331	3996	991	2288	5978	1989	76
2014	2492	6488	1191	3465	9443	1454	144
Total	6488	6488	2277	9443	9443	3540	87

The greater treatment area included the treatment area as well as the immediate surrounding area (see Fig 1).

### Telemetry and nest data

Sage-grouse females were captured during winter and spring from 2009–2014 in the treatment area and 2010–2014 in the control area using spotlighting techniques [34,35] near leks and wintering habitat with a goal of ~40 radio-collared (22-g VHF radio-collars, model #A4060, Advanced Telemetry Systems, Isanti, MN, USA) females at start of nesting in each of the two areas; 93% of females were captured prior to the onset of nest initiation (1 Apr), and 100% well in advance of median nest initiation (29 Apr). The sample of monitored grouse declined throughout the year between trapping seasons due to mortality and transmitter failure, but the sample was then increased each year to our goal of 40 females per area by the beginning of nesting. Capture and handling methods were approved by the University of Idaho Animal Care and Use Committee (Protocol Number: 2012–16) and the Oregon Department of Fish and Wildlife. While transmitters were equipped with 8-hr mortality switches, encounters generally consisted of telemetry locations acquired by approaching to within 30 m of the birds without flushing. For the survival analysis, we tried to encounter each bird 1–2 times per week during February–July and 1–4 times per month during August–January. For the nest survival analysis, we monitored radio-marked females twice per week during the potential nesting seasons from 2011–2014. When a female was observed in the same place on two consecutive locations, she was then observed visually, without flushing, to verify nesting. Nests were subsequently monitored twice per week until incubation was terminated (e.g., hatched, depredated). All nests were included as independent replicates for the analyses even though some females nested in multiple years (n = 33) or re-nested after failure during the same year (n = 19). Although some autocorrelation in these instances likely exists, we believe interannual variation, such as weather patterns, was more likely to increase independence, and thus, including all data was more beneficial than disregarding these potential pseudo-replicates. However, we assessed nest survival structures including within-year nest type (i.e., first nest or renest) to determine and control for the underlying structure.

### Female survival BACI analysis

We estimated female sage-grouse survival with the nest survival model in MARK [36,37] using the RMark package [38] as an interface within R 3.1.2 [39]. Because the control area data collection started in the fall of 2010, we used treatment area data also beginning at this time. As treatments generally concluded at the start of the sage-grouse nesting season (March or April), we defined years biologically as April to March. Survival histories were relatively complete from April to July (1–2 encounters per week), but were sparse during the rest of the year (1–2 encounters per month), and we therefore, pooled samples into monthly encounter histories throughout the year.

We initially compared monthly, seasonal, and annual survival models with Akaike’s information criterion corrected for small sample sizes (AICc) [40] to select a within-year temporal structure to control for survival heterogeneity in the subsequent conifer removal analysis (Table 2). We assessed 4 seasonal structures including 1) breeding where April–July was separated from the rest of the year, 2) seasonal where 2 intervals were used: April–September and October–March, 3) seasonal where 3 intervals were used: April–July, August–November, and December–March, and 4) seasonal where 4 intervals were used: April–June, July–September, October–December, and January–March.

**Table 2:** Survival models assessed for female sage-grouse.

	Model^1^	k	AICc	ΔAICc	Weight	Deviance
A)	Breeding	2	948.25	0.00	0.65	944.25
	Season3	3	950.25	2.00	0.24	944.24
	Season4	4	953.54	5.28	0.05	945.52
	FYear + Breeding	6	954.05	5.80	0.04	942.02
	FMonth	12	955.88	7.63	0.01	931.75
	FMonth + Breeding	13	957.91	9.66	0.01	931.75
	Season2	2	958.16	9.91	0.00	954.15
	FMonth + FYear	16	962.31	14.06	0.00	930.08
	Null	1	965.00	16.75	0.00	963.00
	FYear	5	970.28	22.03	0.00	960.26
B)	Breeding + TreatBreedingTrend	3	948.60	0.00	0.27	942.59
	Breeding + TreatTrend	3	948.83	0.23	0.24	942.82
	Breeding + Trend	3	949.46	0.86	0.18	943.45
	Breeding + Area	3	949.97	1.37	0.14	943.96
	Breeding + BeforeAfter	3	950.16	1.56	0.12	944.15
	Breeding + Area + Trend + Area × Trend	5	952.77	4.17	0.03	942.74
	Breeding + Area + BA + Area × BA	5	953.87	5.27	0.02	943.84

(A) Overall temporal structure of survival assessed for use in conifer removal experiment assessment in B. (B) Comparison of treatment effect models with non-treatment effect models.

^1^ Breeding = 2 seasons: April–July and August–March. Season2 = 2 seasons: April–September, October–March. Season3 = 3 seasons: April–July, August–November, and December–March. Season4 = 4 seasons: April–June, July–September, October–December, January–March. FYear = categorical year. FMonth = categorical month. Area = treatment (conifer removal) area or control area. Trend = continuous year trend. BA = categorical before and after treatments began. TreatBreedingTrend = trend in treatment area breeding seasons, constant during nonbreeding and in control area. TreatTrend = trend in all season in treatment area, constant in control.

In an environmental impact analysis, the important parameter is the impact × area (e.g., control or treatment) interaction and the additive effects are unimportant [31]. The impact can be represented as a before-after impact factor or continuous time variable [30]. Because conifer removal accumulated over time, in our analysis we assessed 2 BACI models with different measures of impact including interactions between study area and 1) before-after impact using April 2012 as the split and 2) linear time trend (Table 2). We also assessed a model where the control area had constant survival and the treatment area had an annual trend and a model with a trend among breeding periods (nesting and early brood rearing; April–July) but constant survival in nonbreeding periods. We also assessed models with just year, study area, or before-after to use as null models. We compared all models with AICc and model weights [40].

To assess the magnitude of treatment effect on survival for the best treatment effect trend model, we calculated the annual survival and the standardized difference between the control and treatment areas and calculated the 85% confidence intervals [41]. Additionally, we estimated independent survival trends in the treatment and control areas using the annual trend BACI model.

### Nest survival BACI analysis

We estimated daily nest survival with the nest survival model in MARK [36,37] using the RMark package [38] as an interface within R 3.1.2 [39]. For the BACI analysis, we used only nests from 2011 onward because this was the first nest season of data collection in the control area.

We initially compared weekly, biweekly, and monthly nest survival models along with female age (juvenile or adult), nest initiation, continuous time trend within year, and nest type (first or renest) with AICc [40] to select a structure to control for survival heterogeneity in the subsequent conifer removal analysis (Table 3). Similar to the female survival treatment effect models, we assessed BACI models with interactions between study area and 1) before-after impact using 2012 as the split and 2) linear time trend (Table 3). We also assessed a model where the control area had constant survival and the treatment area had an annual trend. We assessed models with just year, study area, or before-after to use as null models. We compared all models with AICc and model weights.

**Table 3:** Nest survival models assessed for sage-grouse.

	Model^1^	k	AICc	ΔAICc	Weight	Deviance
A)	Null	1	665.52	0.00	0.34	663.52
	NestType	2	667.07	1.55	0.16	663.07
	Time	2	667.37	1.85	0.14	663.36
	Initiation	2	667.38	1.86	0.13	663.38
	HenAge	2	667.47	1.96	0.13	663.47
	FMonth	3	668.94	3.43	0.06	662.94
	FBiweek	7	669.80	4.28	0.04	655.77
	FWeek	13	678.21	12.69	0.00	652.11
B)	BA	2	660.71	0.00	0.48	656.70
	Trend	2	662.37	1.66	0.21	658.37
	TreatTrend	2	663.30	2.59	0.13	659.30
	Area + BA + Area × BA	4	663.92	3.22	0.10	655.91
	Area + Trend + Area × Trend	4	664.87	4.16	0.06	656.86
	Area	2	667.15	6.44	0.02	663.15

(A) Overall structure of survival assessed for use in conifer removal experiment assessment in B. (B) Comparison of treatment effect models with non-treatment effect models.

^1^ NestType = 1^st^ nest or renest. Time = trend within nesting period. HenAge = adult or juvenile. Initiation = date the nest was first located. FMonth = categorical month within nesting period. FBiweek = categorical biweekly intervals within nesting period. FWeek = categorical weekly intervals within nesting period. Area = treatment (conifer removal) area or control area. Trend = continuous year trend. BA = categorical before and after treatments began. TreatTrend = trend in treatment area, constant in control area.

To assess the magnitude of treatment effect on nest survival for the best treatment effect trend model, we calculated the total nest survival (using a 37-day incubation period) [42,43] and the standardized difference between the control and treatment areas and calculated the 85% confidence intervals. Additionally, we estimated independent nest survival trends in the control and treatment areas using the annual trend BACI model.

### Projected population growth rates

Nest success and female survival are two of the most important demographic vital rates among sage-grouse [29,44]. As management actions targeting demographics ultimately aim to influence population growth, we used a model-based approach to assess the influence of treatment effects on asymptotic population growth rate (λ). Specifically, we generated two-stage (yearling and adult) female-based population projection models employing the formulation developed by Taylor et al. [29], using vital rate estimates for the control and treatment areas from the most supported models. Projection matrices were based on a pre-breeding birth-pulse census with annual time steps composed of elements for stage-specific fecundity and survival. Because our interests for demographic estimation lay solely in nest and annual female survival, we did not track lower level components for fecundity including nest initiation rate, clutch size, hatching rate, and chick (hatch to 35 d) and juvenile (35 d to following breeding season) survival. For these vital rates, we used mean estimates from a recent rangewide meta-analysis [29].

Accounting for variation among vital rates is critical to analyses of population growth [29,44]. Therefore, we generated beta distributions (using stretched-beta distribution for clutch size) to represent the central tendency and variance of each vital rate. Variance components for vital rates estimated by Taylor et al. [29] include only process variance, which was estimated using mixed effects generalized linear models. Our nest and annual female survival variance components are conflated with both process and sampling variance such that estimated variation in growth rates is likely inflated. However, we believe this method still provides an informative comparison of growth rates between our control and treatment areas. We generated 10,000 population projection matrices for both the control and the treatment distributions and calculated λ as the dominant eigenvalue across projection matrices.

## Results

### Female survival BACI

We captured 108 and 116 female sage-grouse from 2010–2014 in the control and treatment areas, respectively. Five birds were removed from the analysis because they left the study area or were not encountered after capture resulting in a sample of 107 and 112 in the control and treatment areas, respectively. Of the 219 birds, 141 died during the study and 78 were right censored because the transmitter failed or the study concluded in August 2014. The breeding season model (breeding: April–July, nonbreeding: August–March) was the best temporal structure to estimate survival with a weight of 0.65 (Table 2A) and was used as the base structure in the subsequent conifer removal analysis.

All treatment models had higher AICc values than the base model; however, two of these models differed from the best supported model by only a fractional amount (ΔAICc = 0.35, 0.58) indicating some support for treatments improving survival. The treatment breeding trend model had the lowest AICc and therefore had the greatest support (model weight: 0.27), but the model with a trend throughout the year in the treatment area was highly competitive (model weight: 0.24; Table 2B). The two interactive models, annual trend and before-after, had low support (models weights: 0.03 and 0.02) but were within 6 AICc units and should not be discounted.

**Fig 3:**
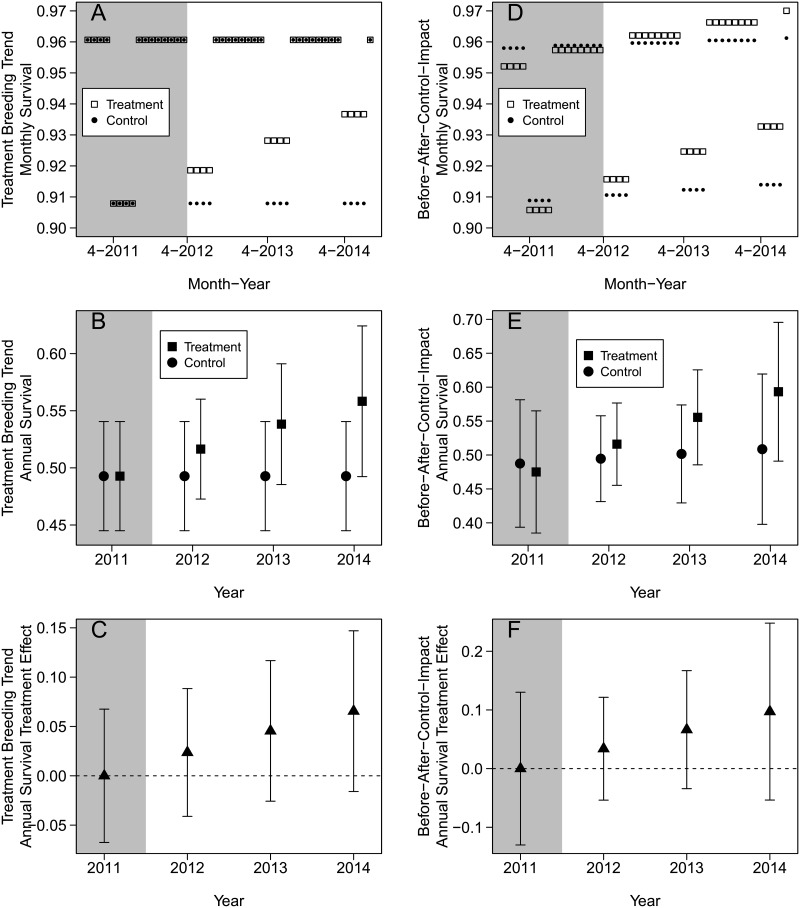
Landscape-scale conifer removal effects on female sage-grouse survival estimates. (A–C) Treatment breeding trend model. (D–F) Time-trend before-after-control-impact model (Trend × Area). Error bars represent 85% CIs. Shaded area represents before conifer removal. Horizontal dashed line represents no treatment effect. (A and D) Monthly survival estimates in the control and treatment (conifer removal) areas. (B and E) Estimated annual survival in the control and treatment areas. (C and F) Difference in annual estimates between control and treatment areas. A positive slope in C and F indicates potential treatment effect.

The survival estimates of the top model showed a positive trend in the breeding season through time in the treatment area (Figs 3A and 2B), but the confidence interval overlapped zero (β = 0.135 [85% CI: -0.019–0.290]). The confidence intervals were large relative to the effect, but the estimated treatment effect in annual survival was a 6.6% (85% CI: -1.6–14.7) increase in the treatment area relative to the control area by 2014 (Fig 3C).

The annual trend interactive model (Trend × Area) also showed a positive trend though time (Figs 3D and 2E), but the confidence interval of the interaction overlapped zero (β = 0.101 [85% CI: -0.121–0.323]). The confidence intervals were large relative to the effect, but the estimated treatment effect in annual survival was a 9.7% (85% CI: -5.4–24.8) increase in the treatment area relative to the control area by 2014 (Fig 3F).

### Nest survival BACI

We located 109 and 123 nests from 2011–2014 in the control and treatment areas, respectively. Seven nests were removed because they were abandoned and we suspected observer influence. Of the remaining, 116 nests were successful and 109 were unsuccessful. The null model was the best temporal structure with a model weight of 0.34 (Table 3A) and was used as the base structure in the conifer removal analysis.

The non-treatment time trends, before-after and yearly, had the lowest AICc values (model weight 0.48 and 0.21; Table 3B). The most supported treatment effect model was the trend in the treatment area (model weight: 0.13), while the interactive treatment models also garnered some support (model weight: 0.10 and 0.06; Table 3B).

**Fig 4:**
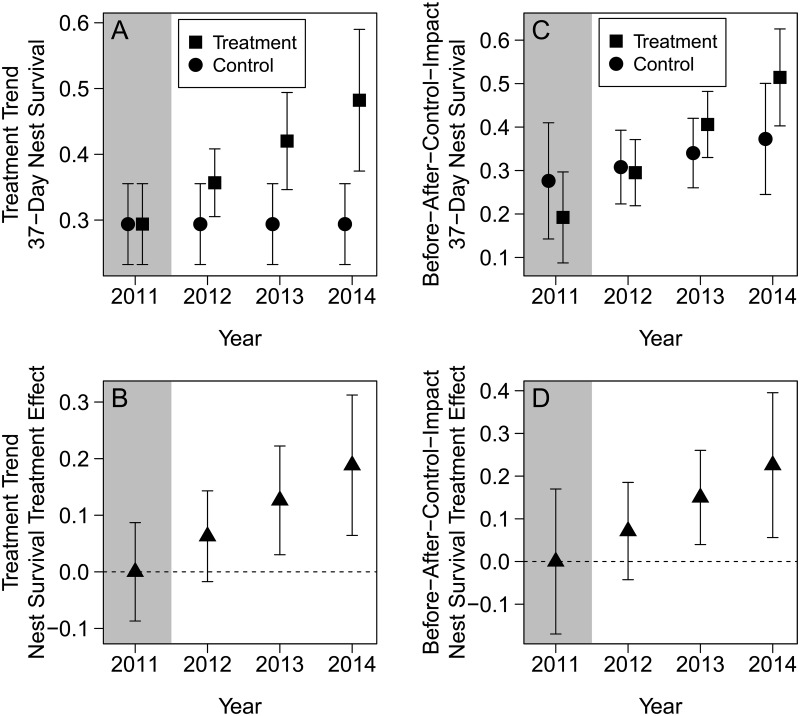
Landscape-scale conifer removal effects on sage-grouse nest survival estimates. (A–B) Treatment trend model. (C–D) Time-trend before-after-control-impact model (Trend × Area). Error bars represent 85% Cis. Shaded area represents before conifer removal. Horizontal dashed line represents no treatment effect. (A and C) 37-day nest survival estimates in the control and treatment (conifer removal) areas. (B and D) Difference in 37-day estimates between control and treatment areas. A positive slope in B and D indicates potential treatment effect.

The nest survival estimates of the best treatment model showed a positive trend through time in the treatment area (Fig 4A), and the confidence interval of the slope did not overlap zero (β = 0.175 [85% CI: 0.049–0.301]). The estimated treatment effect in 37-day nest survival was a 18.8% (85% CI: 6.4–31.2) increase in the treatment area relative to the control area by 2014 (Fig 4B).

The nest survival estimates of the annual trend interactive model (Trend × Area) showed a positive trend through time in the treatment area relative to the control area (Fig 4C), and the confidence interval of the interaction slope slightly overlapped zero (β = 0.218 [85% CI: -0.055–0.490]). The estimated treatment effect in 37-day nest survival was a 22.3% (85% CI: 5.6–39.5) increase in the treatment area relative to the control area by 2014 (Fig 4D).

### Population growth rates

For nest survival, we used estimates from the trend model of 0.294 (85% CI: 0.232–0.355) for the control area and 0.482 (85% CI: 0.375–0.590) for the treatment area. Because our model selection approach did not identify female age as an important source of variation, we applied the estimates across both stages (juvenile and adult). Similarly, for female survival, we applied the final year’s estimates of the breeding trend model of 0.493 (85% CI: 0.445–0.541) for the control area and 0.558 (85% CI: 0.492–0.624) for the treatment area. Results from the simulation revealed a mean population growth rate of 0.85 (85% CI: 0.71–1.03) for the control area and 1.10 (85% CI: 0.89–1.39) for the treatment area.

## Discussion

Our study is one of the first to link sage-grouse demographic rates with conifer removal treatments. At the landscape scale, we observed support for potentially increased female survival and nest survival, which are two of the most important demographic parameters affecting population growth [29]. Applying demographic estimates to population models suggested that treatments increased the population growth by 25% relative to the control from slightly a declining to an increasing population. Inference from population projection matrices relies on the assumption that unmeasured vital rates (e.g., chick survival) in our study area were similar to those from the rangewide meta-analysis. As our nest and female survival estimates were within the range of the meta-analysis estimates, we suspect that other vital rates did not vary substantially from those found in the literature. Furthermore, if conifer removal benefits the other vital rates as well, we might expect an even greater increase in λ. While further monitoring is needed, our results indicate potential positive effects on sage-grouse population growth which corresponds to the desired outcome of wildlife habitat restoration [27]. Baruch-Mordo et al. [20] found conifer expansion decreased lek occupancy, which is a proxy for population size, and recommended conifer removal near leks, while Coates et al. [19] suggested that decreases in populations are in part due to decreased female survival caused by increased conifer abundance. Sanford et al. [45] observed increased nest and brood success near conifer removal areas, but we provide the first empirical evidence that conifer removal may lead to increases in population-level demographic rates.

We speculate the mechanisms affecting survival in our study include reduced avian predator populations and hunting efficiency as well as increased availability of high quality habitat [32]. Avian predators are important sources of mortality for sage-grouse adults and eggs throughout their range [46] with corvids focusing on eggs [47] and raptors largely preying on adults [48]. Avian predator distribution and abundance are limited by nesting and perching sites [49,50], and eliminating these sites may be beneficial in reducing depredation rates. Additionally, changes in available habitat as a result of conifer removal may also impact survival. Because sage-grouse select seasonal habitats and nest sites in areas susceptible to conifer invasion [19,32], conifer removal in those areas increases the availability of higher quality habitat [32]. Compared to other sagebrush habitat types, increased nest survival has been observed in mountain big sagebrush [32], which is also more susceptible to conifer invasion than low sagebrush or Wyoming big sagebrush habitat [4] implying reductions in nest survival as conifer expansion and infill continues, but also opportunities for effective restoration.

At the landscape scale that we assessed, we observed benefits of conifer removal. However, at a smaller extent, conifer removal may affect individuals differently. Coates et al. [19] documented a potential ecological trap at low conifer canopy cover, while Severson [32] suggested that abrupt edges between woodlands and open sagebrush areas may pose a risk for some individuals. However our study, along with those of Baruch-Mordo et al. [20] and Sandford et al. [45], implies that landscape-scale conifer removal benefits sage-grouse populations as a whole. More research is needed at finer scales to fully assess the effect of conifer removal on individual grouse to better inform management planning and maximize population-level benefits.

Although management of tree invasions has long been suggested for conserving prairie and sage-grouse [28,50–52], few studies have actually assessed those effects [18,53]. Many studies have documented negative effects of woody encroachment on prairie grouse habitat selection [54–57], but our study represents a major step forward in evaluating the effects of a landscape-scale habitat restoration on landscape-scale demographics resulting in population growth. While our results generally indicate positive outcomes of conifer removal on sage-grouse, much remains to be learned. Further monitoring at this site, as well as other sites throughout the sage-grouse range, will be necessary to understand long-term effects of conifer removal on survival at both the landscape scale and to individual birds. Future analyses should include other demographic parameters (e.g., brood survival) and response in long-term population trends from lek counts.

Sage-grouse have become a primary catalyst for large-scale investment in sagebrush habitat conservation [18], and our findings have ramifications for other ecosystem services stemming from conifer removal as well. Holmes et al. [58] found abundance of sagebrush-obligate songbirds of conservation concern, such as Brewer’s sparrow (*Spizella breweri*), increased in response to conifer removal primarily conducted to benefit sage-grouse. At regional scales, 85% of conifer removal conducted through the Sage Grouse Initiative coincided with Brewer’s sparrow abundance centers suggesting that considerable benefits for less well-known wildlife species may be accruing through continued investment in conifer removal for sage-grouse [59]. Conifer removal preserves basic sagebrush ecosystem functions by enhancing resilience to wildlife and resistance to cheatgrass (*Bromus tectorum*) [60,61], extending soil water availability for plant growth [62], and benefiting water storage and delivery [63]. From a socio-economic standpoint, sustainability and profitability of livestock ranching, the predominant land use in the West, also improves with conifer removal by maintaining forage production [64].

## Management implications

Conifer removal appears to benefit survival of female sage-grouse and their nests, which could yield increases in population growth through time. Landscape-scale removals may be important in project planning; in our treatment area, conifer treatments totaled 6488 ha with individual cut areas averaging 87 ha and cumulatively covering ~20% of our study area. While the scale of restoration necessary to have a positive effect is still unknown, given the ecology of this landscape species, effort should be made to maximize the size of treatments. Additionally, we recommend maximizing the contiguous area of conifer removal and existing treeless habitat as opposed to patchy treatments.

## Supporting information

S1 Table Monthly survival for female sage-grouse estimated from treatment breeding trend model and BACI (Area × Trend interaction) model. (XLSX)

S2 Table Daily nest survival for age-grouse estimated from treatment trend model and BACI (Area × Trend interaction) model. (XLSX)
